# Association analysis of genetic and environmental risk factors in the cuticular drusen subtype of age-related macular degeneration

**Published:** 2012-08-18

**Authors:** Johannes P.H. van de Ven, Dženita Smailhodzic, Camiel J.F. Boon, Sascha Fauser, Joannes M.M. Groenewoud, N. Victor Chong, Carel B. Hoyng, B. Jeroen Klevering, Anneke I. den Hollander

**Affiliations:** 1 Department of Ophthalmology, Radboud University Nijmegen Medical Center, Nijmegen, the Netherlands; 2 Department of Vitreoretinal Surgery, Center for Ophthalmology, University of Cologne, Cologne, Germany; 3 Department of Epidemiology, Biostatistics, and HTA, Radboud University Nijmegen Medical Center, Nijmegen, the Netherlands; 4 Oxford Eye Hospital, University of Oxford, England, Oxford, UK; 5 Department of Human Genetics, Radboud University Nijmegen Medical Center, Nijmegen, the Netherlands

## Abstract

**Purpose:**

To assess the association of gender, cigarette smoking, body-mass index, and nine genetic risk variants with cuticular drusen (CD), a well recognized subtype of age-related macular degeneration (AMD).

**Methods:**

A total of 757 patients with AMD, including 217 patients with CD, and 553 control individuals were interviewed with a questionnaire and underwent an ophthalmic examination. Venous blood samples were obtained for genomic DNA extraction, and genotyping was performed of single nucleotide polymorphisms previously associated with AMD. Odds ratios were calculated for patients with CD, using unaffected control individuals as a reference. Furthermore, odds ratios in patients with CD were compared to those in patients with “non-CD” AMD.

**Results:**

The CD subtype of AMD was significantly associated with current smoking as well as variants in the complement factor H (*CFH*), age-related maculopathy susceptibility 2 (*ARMS2*), complement factor B/complement component 2 (*CFB/C2*), complement component 3 (*C3*), and apolipoprotein E (*APOE*) genes. In patients with CD, the association with the *CFH* Y402H risk allele was significantly higher (p=0.022), whereas the association with current smoking was significantly lower (p<0.001) than in the heterogeneous group of patients with “non-CD” AMD.

**Conclusions:**

The AMD subtype of CD was associated with previously identified genetic AMD risk factors. However, the association with the *CFH* Y402H risk allele appeared to be stronger, whereas the association with smoking was less pronounced when compared to AMD as a whole. This study suggests a more important role for genetic factors than environmental factors in the development of this well defined subtype of AMD. These findings stress the importance of detailed phenotyping in AMD to identify homogeneous AMD subtypes, which may be associated with different risk factors and disease mechanisms. Such studies will improve the accuracy of predictive models and the effectiveness of preventive and therapeutic options in AMD.

## Introduction

Age-related macular degeneration (AMD) is the most common cause of irreversible and progressive visual loss among the elderly in the Western world [1,2]. The abnormalities of this disorder range from discrete drusen deposits and pigmentary changes in early AMD to geographic atrophy and/or choroidal neovascularization (CNV) in the advanced forms.

AMD is a clear example of a multifactorial disease, and a wide variety of risk factors have been associated with the development and progression of AMD. Advanced age, female gender, cigarette smoking, and a high body-mass index (BMI >30) have been reported as the most consistently reproducible demographic and environmental risk factors in AMD [3-7]. Familial aggregation analyses and twin studies have provided clear evidence of heritability, and more recently strong associations were found with the Y402H (rs1061170) polymorphism in the complement factor H (*CFH*) gene and with the A69S (rs10490924) polymorphism in the age-related maculopathy susceptibility 2 (*ARMS2*) gene [8-12]. These two allelic variants contribute to late AMD in more than 80% of cases [13,14]. Other genes that harbor established risk variants for AMD include the complement factor B (*CFB*), complement component 2 (*C2*), complement component 3 (*C3*), complement factor I (*CFI*), and the apolipoprotein E *(APOE*) genes [15-22].

The fact that AMD is highly heterogeneous in its clinical presentation is well recognized. Nevertheless, most studies reporting on the influence of environmental and genetic risk factors analyzed the AMD phenotype as a whole, without attempting to determine these risk factors in more homogeneous subtypes of the disorder. Based on the clinically observed abnormalities, several subtypes of AMD may be recognized, including polypoidal choroidal vasculopathy, retinal angiomatous proliferation, and cuticular drusen (CD) [23-29]. The latter, formerly known as basal laminar drusen [30], is characterized by the fundoscopic findings of innumerable, small (25 µm to 75 µm), uniformly sized, round drusen [31]. Most commonly appearing in early adulthood, these drusen are easy visualized with fluorescein angiography (FA). In more advanced stages, the multitude of drusen produce a typical “stars-in-the-sky” appearance in early phases of the angiogram [32]. Researchers have estimated that the CD phenotype comprises approximately 10% of the AMD spectrum [29].

In the present study, we investigated whether the AMD subtype of CD displays different environmental and genetic risk factors than AMD as a whole.

## Methods

### Subjects

In this study, we evaluated a total of 757 unrelated patients with AMD, including 217 patients with CD, and 553 control individuals. All subjects were retrieved from the European Genetic Database (EUGENDA), a multicenter database for clinical and molecular analysis of AMD. In the current study, only Caucasian participants from the Nijmegen (the Netherlands) area participated.

Before being enrolled in EUGENDA, all subjects were interviewed with a questionnaire to document their medical history and lifestyle habits, such as BMI and smoking status. Subjects who reported a kidney disease were excluded from the study to preclude including patients with membranoproliferative glomerulonephritis (MPGN) type II. Pupillary dilatation was achieved with topical 1.0% tropicamide and 2.5% phenylephrine before retinal imaging. Digital non-stereoscopic 30° color fundus photography was performed with a Topcon TRC 50IX (Topcon Corporation, Tokyo, Japan). In addition, patients with AMD received FA and high-resolution Fourier-domain optical coherence tomography (FD-OCT), performed with a combined confocal scanning laser ophthalmoscope/FD-OCT device (SPECTRALIS, Heidelberg Engineering, Heidelberg, Germany).

Color fundus photographs of both eyes of all cases were evaluated by two independent reading center graders according to the standard protocol of the Cologne Image Reading Center and Laboratory (CIRCL). AMD was defined by using international standards as described previously [33]. Individuals of similar age as the AMD cases and who exhibited no signs of AMD in either eye were collected as controls. The CD subtype was defined as a symmetric distributed pattern between both eyes of at least 50 scattered, uniformly-sized, small (25 µm to 75 µm) hyperfluorescent drusen on FA in each eye, of which a minimum of 20 drusen are located outside the Wisconsin age-related maculopathy grading template [34]. After the grading was completed, the AMD cohort was divided into a cohort of patients with the AMD subtype of CD and a group of patients with “non-CD” AMD.

This study was reviewed and approved by the local institutional review boards and adhered to the tenets of the Declaration of Helsinki. Written informed consent was obtained from all individuals before they participated in the study.

### Single nucleotide polymorphism genotyping

Genomic DNA was isolated from peripheral blood leukocytes using standard techniques and stored at −20 °C. Genotyping of single nucleotide polymorphisms (SNPs) in the *CFH* (rs1410996), *ARMS2* (rs10490924), *CFB* (rs4151667), *C2* (rs9332739), *C3* (rs2230199), *CFI* (rs10033900), and *APOE* (E2 allele; rs7412 and E4 allele; rs429358) genes in the “non-CD” AMD, CD, and control cohorts were performed as previously described [35]. The *CFH* variant Y402H (rs1061170) was analyzed with direct sequencing of PCR products using forward primer 5′-TCA TTG TTA TGG TCC TTA GG-3′ and reverse primer 5′-AAA GAC ATG AAC ATG CTA GG-3′. These nine SNPs were selected because they were previously associated with AMD [8-12,15-22]. Fourteen percent of the genotypes were done in duplicate, resulting in a concordance of ≥99.9%.

### Statistics

Genotype frequencies in the control individuals were tested for Hardy–Weinberg equilibrium. Baseline and clinical characteristics were analyzed with standard descriptive statistics, and differences in gender, smoking status, and BMI were analyzed with a multivariate logistic regression analysis to adjust for the covariates age, gender, BMI, and smoking status where applicable. Subsequently, to study the associations of allele frequencies for AMD-associated SNPs among the “non-CD” AMD cohort, the CD cohort, and the controls, a multivariate logistic regression analysis was performed to adjust for the covariates age, gender, smoking status, and BMI. The differences between the three cohorts are presented as odds ratios (ORs) with 95% confidence intervals (95% CIs).

Data analysis was performed using SPSS software, version 18.0 (SPSS Inc., Chicago, IL). The reported p values are two-sided, and a value of < 0.05 was considered statistically significant.

## Results

Baseline demographics and risk allele frequencies of the “non-CD” AMD (n=540), CD (n=217), and control (n=553) cohorts are depicted in Table 1 and Table 2. The mean age was 76.7 years (range 55–94; standard deviation [SD] 7.4) in the “non-CD” AMD cohort, 69.3 years (range 50–91; SD 10.4) in the CD cohort, and 73.1 years (range 55–92; SD 6.3) in the controls.

**Table 1:** Demographics in “non CD” AMD, CD and control individuals

			**“non CD” AMD/controls**			**CD/controls**		**CD/”non CD” AMD**	
	**Controls**	**“non CD” AMD**	**p-value**	**OR (95-C.I.)**	**CD**	**p-value**	**OR (95-C.I.)**	**p-value**	**OR (95-C.I.)**
N	553	540			217				
Mean age (SD)	73.1 (6.25)	76.7 (7.42)			69.3 (10.40)				
** Gender**									
Male (%)	242 (43.8%)	210 (38.9%)	0.040*	1.33 (1.01–1.75)*	77 (35.5%)	0.086*	1.36 (0.96–1.92)*	0.232*	1.27 (0.86–1.88)*
Female (%)	311 (56.2%)	330 (61.1%)			140 (64.5%)				
** BMI**									
<25 (%)	242 (43.8%)	224 (41.5%)	Ref.		95 (43.8%)	Ref.		Ref.	
25–30 (%)	249 (45.0%)	235 (43.5%)	0.169^†^	1.21 (0.92–1.59)^†^	92 (42.4%)	0.616^†^	0.92 (0.65–1.30)^†^	0.079†	0.71 (0.49–1.04)^†^
>30 (%)	62 (11.2%)	81 (15.0%)	**0.027^†^**	**1.58 (1.05–2.36)^†^**	30 (13.8%)	0.635^†^	1.13 (0.68–1.89)^†^	0.079†	0.62 (0.36–1.06)^†^
**Smoking status**									
Never (%)	257 (46.5%)	230 (42.6%)	Ref.		99 (45.6%)	Ref.		Ref.	
Past (%)	272 (49.2%)	230 (42.6%)	0.685‡	1.06 (0.80–1.40)‡	96 (44.2%)	0.977^‡^	1.01 (0.71–1.43)^‡^	0.845‡	0.96 (0.64–1.44) ^‡^
Current (%)	24 (4.3%)	80 (14.8%)	**2.4x10^–11‡^**	**5.97 (3.54–10.09)^‡^**	22 (10.1%)	**0.032^‡^**	**2.06 (1.07–4.00)^‡^**	**2.1x10^–4‡^**	**0.32 (0.17–0.58) ^‡^**

Abbreviations: AMD=age-related macular degeneration; CD=cuticular drusen; OR=odds ratio; CI=confidence interval; BMI=body-mass index; Ref.=reference group. * Adjusted for age, body-mass index and smoking status. † Adjusted for age, gender and smoking status. ‡ Adjusted for age, gender and body-mass index. p-values and ORs printed in boldface indicate significant associations.

**Table 2:** Risk allele frequencies in “non CD” AMD, CD and control individuals

	** **	** **	**“non CD” AMD/controls**		** **	**CD/controls**		**CD/”non CD” AMD**	
	**Controls (%)**	**“non CD” AMD (%)**	**p-value**	**OR (95-C.I.)**	**CD (%)**	**p-value**	**OR (95-C.I.)**	**p-value**	**OR (95-C.I.)**
rs10490924/ ARMS2 A69S	21.9	42.9	**2.0×10^−21^**	**2.94 (2.35–3.67)**	38.3	**1.7×10^−9^**	**2.25 (1.73–2.93)**	0.140	0.83 (0.64–1.06)
rs1061170/ CFH Y402H	38.1	57.7	**1.9×10^−14^**	**2.17 (1.78–2.65)**	64.1	**8.6×10^−16^**	**2.88 (2.23–3.73)**	**0.022**	**1.35 (1.04–1.74)**
rs1410996/ CFH	56.9	74.8	**9.7×10^−15^**	**2.25 (1.83–2.76)**	76.8	**3.0×10^−11^**	**2.53 (1.93–3.33)**	0.209	1.20 (0.90–1.61)
rs9332739/ C2 E318D	4.7	2.5	**0.004**	**0.47 (0.28–0.79)**	1.9	**0.016**	**0.38 (0.17–0.83)**	0.744	0.86 (0.34–2.15)
rs2230199/ C3 R102G	20.8	27.4	**0.001**	**1.46 (1.18–1.80)**	27.9	**0.013**	**1.42 (1.08–1.88)**	0.965	1.01 (0.77–1.32)
rs4151667/ CFB H9L	4.9	2.8	**0.006**	**0.49 (0.29–0.82)**	2.3	**0.027**	**0.42 (0.19–0.91)**	0.750	0.86 (0.35–2.14)
rs10033900/ CFI	48.2	51.8	0.101	1.17 (0.97–1.42)	51.5	0.232	1.16 (0.91–1.49)	0.809	1.03 (0.79–1.35)
rs7412/ APOE2	8.0	10.7	0.115	1.29 (0.94–1.77)	12.1	**0.011**	**1.65 (1.12–2.42)**	0.131	1.37 (0.91–2.05)
rs429358/ APOE4	14.4	10.2	0.058	0.74 (0.54–1.01)	11.1	**0.047**	**0.68 (0.46–1.00)**	0.660	0.91 (0.59–1.40)

Abbreviations: AMD=age-related macular degeneration; CD=cuticular drusen; OR=odds ratio; CI=confidence interval; ARMS2=age-related maculopathy susceptibility 2; CFH=complement factor H; C2=complement component 2; C3=complement component 3; CFB=complement factor B; CFI=complement factor I; APOE=apolipoprotein E. Missings in genotypes are <15%. Data are adjusted for age, gender, body-mass index and smoking status. Risk allele frequencies of rs10490924, rs1061170, rs1410996, rs9332739, rs2230199, and rs4151667 are significantly associated with “non CD” AMD compared to controls. Risk allele frequencies of rs10490924, rs1061170, rs1410996, rs9332739, rs2230199, rs4151667, rs7412, and rs429358 are significantly associated with CD compared to controls. The risk allele frequency of rs1061170 is significantly higher in CD compared to “non CD” AMD.

Current smoking showed an association with CD (p=0.032; OR: 2.06; 95% CI: 1.07–4.00), and this association was significantly lower (p<0.001; OR: 0.32; 95% CI: 0.17–0.58) compared to the “non-CD” AMD cohort. Female gender showed a trend (p=0.086), and no association with BMI was found for CD.

All genotype frequencies conformed to Hardy–Weinberg equilibrium in the control cohort. The risk allele frequency of the *CFH* Y402H (rs1061170) variant was 64.1% in the CD cohort, which closely approximates the prevalence reported previously in patients extensively affected with CD [25]. A significantly higher *CFH* Y402H risk allele frequency was found in the CD cohort when compared with the control cohort (p<0.001; OR: 2.88; 95% CI: 2.23–3.73), and when compared to the “non-CD” AMD cohort (p=0.022; OR: 1.35; 95% CI: 1.04–1.74).

The risk allele frequencies of the *ARMS2* (rs10490924), *CFH* (rs1410996), *C3* (rs2230199), and *APOE E2* (rs7412) variants were significantly higher in the CD cohort compared to the control cohort, and the protective allele frequencies of the *C2* (rs9332739), *CFB* (rs4151667), and *APOE E4* (rs429358) variants were significantly lower in the CD cohort compared to the control cohort. These odds ratios were comparable with the “non-CD” AMD cohort, and no significant differences were observed between the CD and “non-CD” AMD cohort for these SNPs. No association with the *CFI* (rs10033900) risk allele was found in the CD cohort.

## Discussion

The clinical spectrum of AMD is broad, and this clinical heterogeneity will influence the results of association studies on demographic, environmental, and genetic risk factors. Improved phenotyping will increase the power of association studies in predictive models for AMD [36,37], and will lead to a better understanding of the pathogenesis of the different AMD subtypes.

In the present study, we focused on CD, a well defined subtype of AMD. The relatively early onset of CD, as well as the observation that the CD phenotype is often clustered in families, implies a greater contribution of the genetic constitution when compared to AMD in general [23,25,29]. This is further supported by our observation that one of the most important environmental risk factors, current smoking, showed a significantly lower association with CD than with “non-CD” AMD. The latter may also imply that the general advice to patients with AMD for cessation of smoking could be of limited effect in individuals with the CD subtype of AMD. However, this does certainly not mean that cessation of smoking should not be encouraged in patients with CD as current smoking could worsen the natural history of the disease.

Genetic evaluation of our CD cohort showed significant associations between this AMD subtype and variants in the *CFH*, *ARMS2*, *CFB*, *C2*, *C3*, and *APOE* genes. Risk alleles of the rs1410996 (*CFH*), rs10490924 (*ARMS2*), rs4151667 (*CFB*), rs9332739 (*C2*), rs2230199 (*C3*), rs7412 (*APOE E2*), and rs429358 (*APOE E4*) SNPs were significantly associated with CD. However, no significant differences for the previously mentioned risk alleles were observed between patients with CD and AMD in general. This suggests that there is a shared genetic background between AMD in general and CD, which has also been described for other AMD subtypes such as polypoidal choroidal vasculopathy and retinal angiomatous proliferation [24,26,28,38]. A lack of association between the rs10033900 (*CFI*) risk allele and CD could be due to insufficient power in our study to detect small effects. However, the debate over whether this variant is associated with AMD continues as conflicting results have been observed [16,17,39]. Additional studies are needed to clarify the nature of the association between AMD and this particular variant near the *CFI* gene.

A previous study of Caucasian patients who were severely affected with CD demonstrated a strong association with the Y402H (rs1061170) variant in the *CFH* gene [25]. Our study shows that, in spite of the various stages of the CD phenotype included in our cohort, patients with CD are 1.35 times more likely to carry the *CFH* Y402H risk allele compared to patients with “non-CD” AMD. This higher allele frequency of the *CFH* Y402H risk allele in patients with CD suggests that activation of the alternative pathway of the complement system may play a larger role in the pathogenesis of the CD phenotype than in the remainder of the AMD phenotypes [40,41]. This is supported by our previous studies that identified rare pathogenic *CFH* mutations in a subset of families with CD [23,29]. These mutations have not been found in patients with AMD who did not display the CD phenotype. In patients with MPGN type II, or dense deposit disease, *CFH* mutations and disturbed serum complement activation levels have also been demonstrated [42]. Remarkably, almost 70% of individuals with MPGN type II develop extensive drusen in a pattern matching that of extensive CD during their second decade of life [43]. In approximately 10% of these patients, CNV and/or central geographic atrophy may develop at a relatively young age [44-46]. These alterations of the complement system may contribute to the relatively early onset of CD.

The increased insights into the mechanisms underlying AMD have led to possible therapeutic options that have recently entered phase 1 clinical trials [47]. One option may be the use of specific anti-inflammatory molecules that block complement activation [47]. These complement inhibitors may especially benefit individuals with the CD subtype of AMD, where complement activation appears more fundamental to the disease process compared to AMD in general.

In conclusion, the analysis of a large cohort of the CD subtype of AMD has revealed that genetic risk factors affecting the complement system are especially prevalent in these patients. In addition, the environmental risk factor of smoking appears less influential than in AMD in general. These findings stress the importance of detailed phenotyping in AMD to identify homogeneous AMD subgroups, which may be associated with different risk factors and disease mechanisms. Such studies may improve the accuracy of predictive models and the effectiveness of preventive and therapeutic options in AMD.
